# Malaria Prevalence among Young Infants in Different Transmission Settings, Africa

**DOI:** 10.3201/eid2107.142036

**Published:** 2015-07

**Authors:** Serign J. Ceesay, Lamine Koivogui, Alain Nahum, Makie Abdoulie Taal, Joseph Okebe, Muna Affara, Lama Eugène Kaman, Francis Bohissou, Carine Agbowai, Benoit Gniouma Tolno, Alfred Amambua-Ngwa, NFaly Bangoura, Daniel Ahounou, Abdul Khalie Muhammad, Stephan Duparc, Kamal Hamed, David Ubben, Kalifa Bojang, Jane Achan, Umberto D’Alessandro

**Affiliations:** Medical Research Council Unit, Fajara, The Gambia (S.J. Ceesay, J. Okebe, M. Affara, A. Amambua-Ngwa, A.K. Muhammad, K. Bojang, J. Achan, U. D’Alessandro);; Institut National de Santé Publique, Conakry, Guinea (L. Koivogui, L.E. Kaman, B.G. Tolno);; Centre de Recherches Entomologiques de Cotonou, Cotonou, Benin (A. Nahum, F. Bohissou, C. Agbowai, D. Ahounou);; National Public Health Laboratory, Kotu, The Gambia (M.A. Taal);; Préfectoral de la santé de Faranah, Guinea (N. Bangoura);; Medicines for Malaria Venture, Geneva, Switzerland (S. Duparc, D. Ubben);; Novartis Pharmaceuticals Corporation, East Hanover, New Jersey, USA (K. Hamed);; London School of Hygiene and Tropical Medicine, London, UK (U. D’Alessandro);; Institute of Tropical Medicine, Antwerp, Belgium (U. D’Alessandro)

**Keywords:** Malaria, young infants, transmission intensity, Africa, transmission settings, epidemiology, parasites, prevalence, anemia

## Abstract

The prevalence and consequences of malaria among infants are not well characterized and may be underestimated. A better understanding of the risk for malaria in early infancy is critical for drug development and informed decision making. In a cross-sectional survey in Guinea, The Gambia, and Benin, countries with different malaria transmission intensities, the overall prevalence of malaria among infants <6 months of age was 11.8% (Guinea, 21.7%; The Gambia, 3.7%; and Benin, 10.2%). Seroprevalence ranged from 5.7% in The Gambia to 41.6% in Guinea. Mean parasite densities in infants were significantly lower than those in children 1–9 years of age in The Gambia (p<0.0001) and Benin (p = 0.0021). Malaria in infants was significantly associated with fever or recent history of fever (p = 0.007) and anemia (p = 0.001). Targeted preventive interventions, adequate drug formulations, and treatment guidelines are needed to address the sizeable prevalence of malaria among young infants in malaria-endemic countries.

## Abstract 2

Preventive measures and treatment guidelines are needed to address the sizeable prevalence of disease in this population.

Infants are thought to be protected against malaria during the first 6 months of life, largely due to the transfer of maternal antibodies (*1*) and the presence of fetal hemoglobin (*2*). Thus, young infants have received little attention in terms of malaria research and treatment guidelines, and this age group has systematically been excluded from clinical trials. As a consequence, young infants are frequently given off-label antimalarial treatments at dosing schedules recommended for older infants and children (*3*). The lack of attention to case management in this age group is a cause of concern and should be addressed, particularly when considering the widespread use of artemisinin-based combination therapies (ACTs) (*4*) and ongoing antimalarial drug development.

The true prevalence of malaria in young infants is not well characterized, yet defining the prevalence is critical, especially in light of ongoing epidemiologic shifts in populations at risk for malaria (*5*). Data on the prevalence and clinical outcomes of malaria in young infants are limited and contradictory: some studies show minimal risk (*6*–*8*), and others report that the risk for malaria increases in the first months of life, according to the intensity of transmission (*9*). A few reports indicate that the prevalence of disease is higher than previously thought and that, after birth, the period of protection against malaria is shorter than the widely quoted 6 months (*10*,*11*). However, variations in study designs and challenges related to small sample sizes, lack of details regarding quality control, and varied procedures for sample selection make it difficult to interpret findings from earlier studies (*3*). A better understanding of the risk for malaria in early infancy is needed to develop antimalarial drugs and inform policy decisions for this age group (*4*). To improve our knowledge of malaria in young infants, we used standardized methods and more sensitive diagnostics to better characterize the prevalence of malaria among children <6 months of age in different epidemiologic settings.

## Methods

### Study Population and Sampling Design

**Figure 1:**
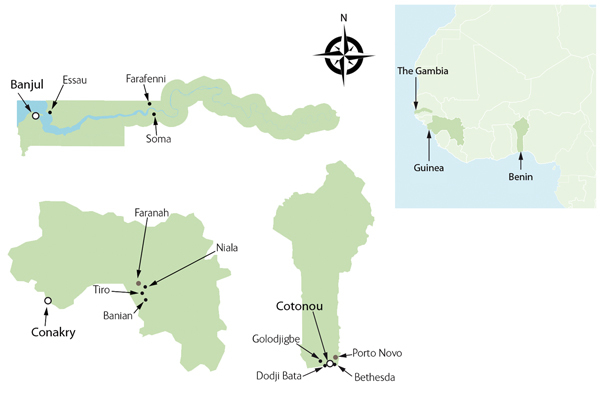
Study sites (arrows) in a study of malaria prevalence among young infants in The Gambia, Benin, and Guinea. Inset shows locations of the 3 countries in western Africa.

This cross-sectional survey was conducted in 3 countries in western Africa: The Gambia, Benin, and Guinea (also known as Guinea Conakry), representing areas of low, moderate, and high malaria transmission, respectively. In each country, regions were selected to represent overall malaria transmission trends and surveys were conducted in the catchment areas of 3 health facilities selected by using simple random sampling. In The Gambia, the Essau and the Soma Major Health Centres and the AFPRC General Hospital in Farafenni were selected out of 6 available sentinel surveillance sites. In the southern part of Benin, Bethesda Hospital and the Dodji-Bata and Golo-Djigbe health facilities were selected from a total of 12 available facilities, and in the Farannah district in Guinea, the Nalia, Tiro, and Banian health centers were also selected from 12 available facilities (Figure 1).

Malaria transmission in The Gambia and Benin is seasonal, occurring during the rainy season, whereas transmission in Guinea occurs year-round. Surveys were timed to coincide with the peak of malaria transmission in each country: October–November 2011 in The Gambia and Guinea; July–August 2012 in Benin. Before conducting the surveys, we explained the study objectives to community members in the catchment areas and obtained community approval. We identified households with infants by reviewing delivery records to detect births in the 6 months before the survey; traditional birth attendants assisted with the reviews. An information sheet explaining the objectives of the survey and the study procedures was then distributed to the parents of the infants. After written informed consent was obtained from parents, identified infants were enrolled in the study. In households with >2 eligible infants, 1 infant was selected by using simple random sampling. Once the index infant was selected, 2 older children (1–9 and 10–15 years of age) living in the same household were also selected by simple random sampling and included in the study with the objective of estimating the force of transmission and differences in the local risk for infection between infants and older children. If children of the required age group were not available within the infant’s household, the nearest households were visited consecutively until eligible children were identified and enrolled.

### Data Collection

Study participants underwent a physical examination; axillary temperature and weight were recorded for each child. Information on the use of bed nets, including long-lasting insecticidal nets, and history of fever in the previous 24 hours was collected by using a structured questionnaire. All study participants had a blood sample collected by finger prick (children 1–15 years of age) or heel prick (infants <6 months of age). A rapid malaria diagnostic test (RDT) (ICT Malaria P.f. Cassette Test [ML01]; ICT Diagnostics, Cape Town, South Africa) was performed, and children who tested positive were immediately treated according to treatment guidelines for the country in which they lived. Hemoglobin concentration was measured by using a HemoCue Hb 301 System (HemoCue AB, Ängelholm, Sweden) according to the manufacturer’s instructions. Thick-film blood slides were stained with 10% Giemsa for 10 min, and the presence of *Plasmodium falciparum* parasites was determined by reading 100 high-power fields under oil immersion. Slides were read independently by 2 microscopists, and parasite density was estimated by counting the numbers of asexual parasites per 200 leukocytes. Results were expressed as the number of parasites per microliter, assuming a total leukocyte count of 8,000 cells/μL. A 20% error check was used to identify discrepancies between slide readers. All discordant results were read by a senior microscopist, and the result was used as the final read. Blood slides from Guinea and The Gambia were read at the Medical Research Council (MRC) Unit in The Gambia; blood slides from Benin were read, following the same protocol, at Entomological Research Centre of Cotonou. The first 99 slides from Benin were read again in The Gambia; results were comparable.

Molecular diagnosis of malaria parasites and speciation of *Plasmodium* species were conducted by using dry blood-spot samples (DBSs) collected on filter paper (Whatman 3MM; Whatman 3 Corporation, Florham Park, NJ, USA). DNA was extracted from 3 disks (6-mm diameter), which had been punched from DBSs by using a QIA X-tractor robot (QIAGEN, Venlo, Limburg, Netherlands) according to the manufacturer’s protocol, and analyzed by using nested PCR as previously described (*12*).

To determine the prevalence of malaria antibodies, we punched disks (6-mm diameter) from DBSs and placed them in 96-well plates. Serum that had been eluted after overnight (18 h) incubation at room temperature in 150 μL of reconstitution buffer (150 μL phosphate-buffered saline/0.05% [vol/vol] Tween 20/0.05% [wt/vol] sodium azide) was used to determine antibodies against the 19-kDa merozoite surface protein 1 (MSP1_19_) by indirect ELISA, as previously described (*13*,*14*). MSP1_19_ used in these assays was obtained from the London School of Hygiene and Tropical Medicine (London, UK).

### Sample Size and Statistical Analysis

The sample size was computed on the lowest expected prevalence of infection, assumed to be 2% in The Gambia. For each country, we estimated that 750 children in each of the 3 age categories would be sufficient to determine the prevalence of malaria. Assuming infants ­<6 months of age made up ≈2%–3% of the total population, the required sample size would be found within a population of ≈40,000 persons.

 Data from the case record forms were double-entered into an OpenClinica database (https://community.openclinica.com/). After being cleaned, the data were analyzed by using Stata Statistical Software, release 12.1 (StataCorp LP, College Station, TX, USA). Baseline data were analyzed by descriptive methods, and summary statistics were presented as means ±SDs for continuous data and frequencies and proportions for categorical data. The χ^2^ test was used to analyze differences in proportions. Two-tailed p values and a 5% significance level were used. Results for infants <6 months of age from all 3 countries were pooled together, and univariate and multivariate logistic regression analyses were performed to determine features associated with malaria in this age group. A forward fitting logistic regression model was used to account for confounders and interaction. The odds for malaria with increasing age within the 0- to 6-month-old age group was determined and presented by country.

For the serologic tests, the distributions of log-transformed antibody titers were fitted as the sum of 2 Gaussian distributions, which were assumed to represent a narrower distribution of seronegative results to the left and a broader distribution of seropositive results to the right. The mean concentration of the seronegative distribution (the distribution with the smallest mean) +2 SDs was considered the seropositivity cutoff (*15*).

### Ethical Considerations

The study was approved by The Gambia Government/MRC Joint Ethics Committee, National Committee of Ethics for Health Research (Benin), and the National Committee of Ethics for Health Research (Guinea). Written informed consent was obtained from the parents of each participant by a signature or thumbprint.

## Results

### Characteristics of the Study Population

A total of 6,761 children were included in the survey: 2,270 from The Gambia, 2,276 from Benin, and 2,215 from Guinea. The number of children categorized by age group, sex, and mean weight by age was comparable between countries (Table 1). Almost 40% (838/2,219) of the infants weighed <5 kg; no difference in weight was seen by country. In The Gambia and Benin, bed net coverage (defined as having slept under a bed net the night before the survey) was >90% in children 0–6 months and 1–9 years of age (Table 1). Conversely, bed net coverage was extremely low across all age groups in Guinea; only ≈30% of children <10 years of age and 14% of children 10–15 years age used bed nets (Table 1). Overall, the prevalence of fever was lower among children 10–15 years than among infants 0–6 months of age. The highest percentage of fevers (48.1%, 359/747] and the lowest mean hemoglobin level (10.0 g/dL [SD 1.7]) were among 1- to 9-year-old children in Guinea.

**Table 1:** Characteristics of children in a study of malaria prevalence among young infants in different transmission settings, Africa

Characteristic	The Gambia, N = 2,270	Benin, N = 2,276	Guinea, N = 2,215
Age groups, no. (%)			
0–6 mo	734 (32.3)	761 (33.4)	724 (32.7)
1–9 y	768 (33.8)	759 (33.3)	748 (33.8)
10–15 y	768 (33.8)	756 (33.2)	743 (33.5)
Sex, no. (%)			
F	1,222 (53.8)	1,189 (52.2)	1,159 (52.3)
M	1048 (46.2)	1087 (47.8)	1056 (47.7)
Mean weight, kg (SD)			
0–6 mo	5.8 (3.6)	5.2 (1.5)	5.5 (2.5)
1–9 y	12.8 (3.6)	14.3 (4.1)	14.4 (4.1)
10–15 y	31.2 (7.5)	30.1 (7.8)	31.6 (7.6)
Bed net coverage, no./no. total (%)*			
0–6 mo	699/727 (96.1)	678/750 (90.4)	225/723 (31.1)
1–9 y	724/765 (94.6)	656/722 (90.9)	222/746 (29.8)
10–15 y	642/752 (85.4)	581/727 (79.9)	103/740 (13.9)
Fever or history of fever, no./no. total (%)†			
0–6 mo	136/732 (18.6)	129/758 (17.0)	282/724 (38.9)
1–9 y	133/768 (17.3)	119/758 (15.7)	359/747 (48.1)
10–15 y	56/760 (7.4)	83/756 (11.0)	221/743(29.7)
Mean hemoglobin level, g/dL (SD)			
0–6 mo	11.8 (2.2)	11.3 (2.0)	12.1 (3.7)
1–9 y	10.9 (1.5)	11.2 (1.4)	10.0 (1.7)
10–15 y	12.4 (1.4)	12.3 (1.6)	11.8 (1.4)

*Defined as having slept under a bed net the night before survey. †History of fever refers to fever in the 24 h before the structured questionnaire was completed.

### Prevalence of Malaria

By all 3 diagnostic methods, malaria prevalence was lowest in The Gambia and highest in Guinea; Benin had intermediate values. In all 3 countries, malaria prevalence was generally lower in infants 0–6 months of age (Table 2). Results from the RDT and microscopy were comparable, although, with 1 exception, the RDT tended to identify more positive samples. The exception was that microscopy showed a much higher prevalence of malaria among young infants in The Gambia (Table 2).

**Table 2:** Prevalence of *Plasmodium* species parasites, by testing method, among children in different transmission settings, Africa

Test method, age group	No./no. total (%)		
	The Gambia, N = 2,270	Benin, N = 2,276	Guinea, N = 2,215
Rapid malaria diagnostic test*			
0–6 mo	3/734 (0.4)	23/761 (3.0)	161/724 (22.2)
1–9 y	11/768 (1.4)	254/759 (33.5)	667/748 (89.2)
10–15 y	35/768 (4.6)	317/756 (41.9)	611/743 (82.2)
Microscopy			
* P. falciparum*			
0–6 mo	25/734 (3.4)	25/761 (3.3)	133/724 (18.4)
1–9 y	8/768 (1.0)	201/759 (26.5)	574/748 (76.7)
10–15 y	21/768 (2.7)	284/756 (37.6)	612/743 (82.4)
*P. falciparum* gametocytes			
0–6 mo	1/734 (0.1)	7/761 (0.9)	61/724 (8.4)
1–9 y	2/768 (0.3)	66/759 (8.7)	138/748 (18.4)
10–15 y	7/768 (0.9)	70/756 (9.3)	91/743 (12.2)
PCR			
*Plasmodium* spp.			
0–6 mo	27/734 (3.7)	78/761 (10.2)	157/724 (21.7)
1–9 y	18/768 (2.3)	243/759 (32.0)	591/748 (79.0)
10–15 y	35/768 (4.6)	324/756 (42.9)	577/743 (77.7)
* P. falciparum*			
0–6 mo	9/734 (1.2)	41/761 (5.4)	139/724 (19.2)
1–9 y	10/768 (1.3)	193/759 (25.4)	531/748 (71.0)
10–15 y	25/768 (3.3)	234/756 (30.9)	502/743 (67.6)
*ICT Malaria P.f. Cassette Test (ML01) (ICT Diagnostics, Cape Town, South Africa).			

By microscopy, all malaria cases identified in children from The Gambia were determined to be caused by infection with *P. falciparum* parasites. In Benin and Guinea, *P. malariae* and *P. ovale* parasite infections were also identified, predominantly as mixed infections. In Guinea, the prevalence of *P. malariae* parasite infections was 0.3% (2/724) in young infants, 12.0% (90/748) in children 1–9 years of age, and 5.8% (43/743) in children 10–15 years of age. Of these infections, 97% (131/135) were mixed infections with *P. falciparum* parasites. The prevalence of *P. ovale* parasite infection in Guinea was 3.1% (23/748) in children 1–9 years of age and 0.9% (7/743) in children 10–15 years of age; no cases were detected among young infants. In Benin, the prevalence of *P. malariae* parasite infection was 0.1% (1/761) in young infants, 1.7% (13/759) in children 1–9 years of age, and 2.8% (21/756) in children 10–15 years of age. In Benin, 34% (12/35) of the infections were mixed *P. malariae* and *P. falciparum* parasite infections. Overall, the mean parasite density per microliter of blood was 371.5 in The Gambia, 1,688.3 in Benin, and 2,037.9 in Guinea.

For the 3 countries, *Plasmodium* spp.–specific PCR also showed a higher prevalence of malaria in all age groups with increasing malaria transmission intensity (Table 2). Prevalence of malaria in infants, as determined by molecular methods, was higher in Guinea (21.7%, 95% CI 18.7%–24.7%) than in Benin (10.2%, 95% CI 8.1%–12.4%) and The Gambia (3.7%, 95% CI 2.3%–5.0%) (Table 2). Species-specific PCR results, compared with microscopy results, showed a lower prevalence of *P. falciparum* parasite infection in children >10 years of age in Benin (30.9% [234/756] vs. 37.6% [284/756]; p = 0.006) and Guinea (67.6% [502/743] vs. 82.4% [612/743]; p<0.0001) (Table 2). However, in The Gambia, species-specific PCR results, compared with microscopy results, showed a lower prevalence of *P. falciparum* infection only in young infants (1.2% [9/734] vs. 3.4% [25/734]; p = 0.005). Gametocyte prevalence by microscopy was lower in infants and increased with age and transmission intensity across the 3 countries (Table 2).

### Prevalence of Malaria Antibodies

Overall, the prevalence of MSP1_19_ antibodies varied from 5.7% among young infants in The Gambia to 45.9% among 1- to 9-year-old children in Guinea (Table 3). Antibody seroprevalence generally increased with age and with transmission intensity across the 3 countries. With the exception of results for children >1 year of age in Guinea, antibody seroprevalence was higher than the prevalence of infection as determined by microscopy (Table 3). For young infants, antibody seroprevalence was also higher than parasite prevalence and increased with transmission intensity from 5.7% in the Gambia to 36.5% in Benin and 41.6% in Guinea.

**Table 3:** Anti–MSP1_19_ antibody seropositivity and *Plasmodium falciparum* parasite prevalence among children in different transmission settings, Africa*

Country, age group	Parasite prevalence, no. (%)†	Antibody seropositive, no. (%)	p value
The Gambia, N = 2,270			
0–6 mo	25 (3.4)	33 (5.7)	0.044
1–9 y	8 (1.0)	53 (7.0)	<0.001
10–15 y	21 (2.7)	57 (8.1)	<0.001
Benin, N = 2,276			
0–6 mo	25 (3.3)	269 (36.5)	<0.001
1–9 y	201 (26.5)	303 (41.5)	<0.001
10–15 y	284 (37.6)	311 (42.5)	0.054
Guinea, N = 2,215			
0–6 mo	133 (18.4)	299 (41.6)	<0.001
1–9 y	574 (76.7)	339 (45.9)	<0.001
10–15 y	612 (82.4)	320 (44.2)	<0.001

*MSP1_19_, 19-kDa merozoite surface protein. †Prevalence as determined by microscopy.

### Malaria in Infants 0–6 Months of Age

The overall prevalence of malaria among infants was 8.2% (183/2,219) as determined by microscopy and 11.8% (262/2,219) as determined by PCR; the prevalence was substantially higher in Guinea than in Benin or The Gambia (Table 2). Mean parasite densities per milliliter of blood were significantly lower in infants than in 1- to 9-year-old children in The Gambia (68/μL [SD 168] vs. 26,708/µL [SD 32,074]; p<0.0001]) and in Benin (6,894/µL [SD 20,567] vs. 12,933/µL [SD 49,895]; p = 0.0021) but not in Guinea (5,725/µL [SD 11,423] vs. 5,479/µL [SD 21,689]; p = 0.89).

Malaria in infants was significantly associated with fever or history of fever in the previous 24 hours (adjusted odds ratio [aOR] 1.65, 95% CI 1.15–2.37; p = 0.007), axillary temperature >37.5°C (aOR 2.07, 95% CI 1.08–3.98; p = 0.029), and anemia (aOR 5.54, 95% CI 3.91–7.84; p = 0.001) (Table 4). Infants weighing <5 kg had significantly higher odds for having malaria (aOR 3.45, 95% CI 2.22–5.26, p = 0.001).

**Table 4:** Characteristics of 0- to 6-month-old infants with malaria in 3 African countries with different transmission settings

Variable	No malaria, n (%), N = 2,036	Malaria, n (%), N = 183	Odds ratio (95% CI)		p value
			Crude	Adjusted*	
Country					
The Gambia	709 (96.6)	25 (3.4)	Reference	Reference	
Benin	736 (96.7)	25 (3.3)	0.96 (0.55–1.69)	0.74 (0.41–1.32)	0.307
Guinea	591 (81.6)	133 (18.4)	6.38 (4.11–9.92)	5.28 (3.30–8.44)	0.001
Sex					
F	1,040 (92.8)	81 (7.2)			
M	996 (90.7)	102 (9.3)	1.31 (0.97-1.78)	1.13 (0.80–1.59)	0.491
Fever or history of fever†					
No	1,573 (94.4)	94 (5.6)			
Yes	458 (83.7)	89 (16.3)	3.25 (2.38–4.44)	1.65 (1.15–2.37)	0.007
Anemia‡					
No	1,724 (95.5)	81 (4.5)			
Yes	312 (75.4)	102 (24.6)	6.96 (5.01–9.67)	5.54 (3.91–7.84)	0.001
Weight, kg					
<5	1,225 (88.7)	156 (11.3)			
>5	811 (96.8)	27 (3.2)	0.26 (0.17–0.40)	0.29 (0.19–0.45)	0.001

*Adjusted for country, sex, fever, anemia, age, and weight. †History of fever refers to fever in the 24 h before the structured questionnaire was completed. ‡Defined as hemoglobin level of <10 g/dL.

**Figure 2:**
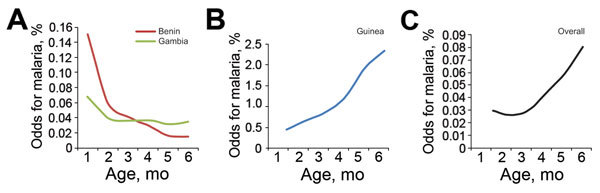
Odds of having malaria, with increasing age, in infants 0–6 months of age in Benin and The Gambia (A), Guinea (B), and in the 3 countries overall (C). Note that the scale of the *y*-axis in panel B differs from that in panels A and C.

**Figure 3:**
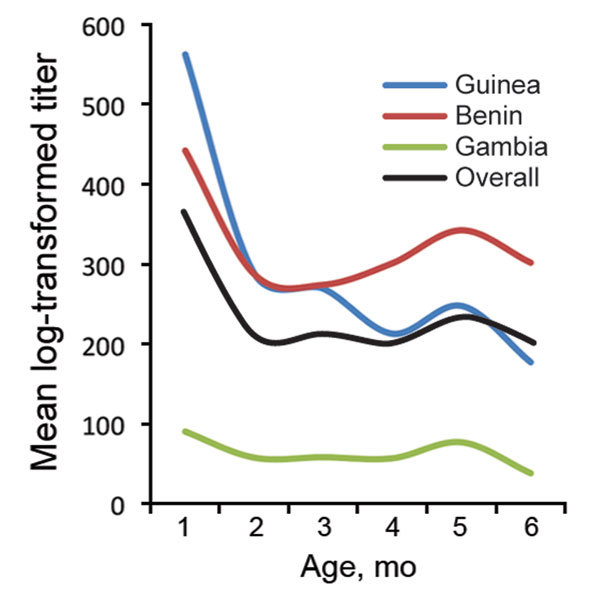
Dynamics of 19-kDa merozoite surface protein antibody titers by infant age in Benin, The Gambia, and Guinea and in the 3 countries overall.

In The Gambia and Benin, the odds for having malaria remained almost stable across the 0- to 6-month-old age group (Figure 2). In contrast, lower odds for having malaria was seen in infants 0–2 months of age in Guinea and markedly increasing odds for having malaria was seen in infants 2–6 months of age. The overall trend was lower odds for malaria in infants 0–3 months of age and subsequently increasing odds for malaria from ≈3 to 6 months of age (Figure 2). In Guinea and Benin, antibody titers were higher early in infancy, declined steadily over the 0- to 4-month age range, and then increased slightly after 4 months of age. Conversely, in The Gambia, infants had lower antibody titers and little evidence of increasing titers over the 0- to 6-month age range (Figure 3).

## Discussion

The prevalence of malaria among infants 0- to 6-months of age was not trivial (range 3.7%–22%, by PCR) and increased with transmission intensity, as documented by the prevalence among older children. This variability in prevalence may be due to differences in transmission intensity but may also be due to differences in the use of preventive measures, as illustrated by the extremely low use of bed nets in Guinea. Such low intervention coverage may be an indicator of weak health systems with limited access to other malaria control interventions (e.g., prompt and efficacious treatment and intermittent preventive treatment for pregnant women), which enable an efficient cycle of malaria transmission in local populations, including infants.

In the low-transmission setting in The Gambia, the risk for malaria did not vary substantially between age groups. This finding was in obvious contrast to those in Benin and Guinea, where prevalence among infants was substantially lower than that among older children. This suggests that in areas where transmission has decreased substantially to low levels, the risk for infection may be shared by the entire population, including infants, and in high-transmission settings, infection in infants may be relatively limited by passively transferred maternal antibodies or possibly by lower attractiveness of infants to mosquitoes (*16*,*17*). The prevalence figures reported are consistent with those of earlier studies, which were limited by smaller sample sizes, different selection criteria, and small geographic areas (*4*,*6*,*11*,*18*–*20*). The results of our surveys in these 3 countries in western Africa provide a regional estimate of the current prevalence of malaria among infants. The survey was conducted by using a relatively large sample size and robust methods of malaria diagnosis, factors that enhance the generalizability of the findings to other settings.

It is not surprising that MSP1_19_ antibody seroprevalence was generally higher than parasite prevalence in young infants: this finding may be a reflection of maternal antibodies passively transferred to the fetus during the last trimester of pregnancy (*21*) and not necessarily a reflection of the infant’s own responses. Prenatal transfer of antibodies may also explain the dynamics of MSP1_19_ antibody titers in young infants. In Guinea and Benin, high titers were observed in children in early in infancy, followed by a rapid decline in mean antibody titers until 4 months of age and then a subsequent slight increase. In contrast, infants in The Gambia had lower antibody titers and little evidence of an increase over the 0- to 6-month age range, indicating no substantial ongoing endogenous antibody production.

*P. falciparum* was the dominant parasite species in all age groups, but a few cases of *P. malariae* and *P. ovale* infection (mostly mixed infections with *P. falciparum* parasites) were found among infants in Guinea and Benin. Therefore, currently available ACTs should suffice for the management of these cases, although failed parasite clearance has been reported in some *P. malariae* and *P. ovale* parasite–infected persons treated with ACTs (*22*).

Malaria in infants was significantly associated with fever or with a history of fever in the 24 hours before the survey, but only 10% of infants with malaria had an axillary temperature >37.5°C at the time of the survey. Although parasite densities in infants were lower than those in older children, about half of the infants with malaria were symptomatic. This finding contrasts with the long-held belief that malaria in young infants is not associated with clinical symptoms (*23*,*24*). The findings from this survey therefore provide evidence that malaria in young infants may be symptomatic and should be evaluated for and treated. In addition, malaria in this age group was significantly associated with anemia, indicating that malaria can have a major negative effect on the health of infants. Other previously reported clinical manifestations (e.g., splenomegaly, hepatomegaly, jaundice, vomiting, diarrhea, poor feeding, restlessness, drowsiness, pallor, respiratory distress, and convulsions) (*25*,*26*) were not consistently documented in this survey. A study systematically investigating for malaria in all 0- to 6-month-old infants attending health facilities in the same areas as this survey has recently been completed and should provide more information on the clinical signs and symptoms of malaria in this age group; that study used RDTs, microscopy, PCR, and hemoglobin measurements.

We have shown that malaria in young infants is not rare, can be symptomatic, and has major health consequences, most notably anemia. Current World Health Organization guidelines recommend the use of ACTs in infants, but they specify that for young infants weighing <5 kg, the available evidence is insufficient to confidently recommend this treatment. Thus, many of the ACTs carry label restrictions saying they should not be used for infants weighing <5 kg (*27*). This restriction is problematic because a substantial proportion (40%) of the infants in these surveys weighed <5 kg and would therefore not meet standard criteria for treatment with ACTs. In addition, there are few pediatric ACT formulations, and the dosing is often difficult. Therefore, data on the efficacy and safety of ACTs in young infants is urgently needed to inform optimal treatment.

The tools used in our study provided comparable estimates of the prevalence of malaria in young infants, with the exception of RDTs, which greatly underestimated the prevalence of malaria in The Gambia, possibly because of the low parasite densities (*28*,*29*). Prevalence estimates determined by microscopy and PCR were surprisingly similar, which may be due to the high sensitivity of microscopy readings conducted in a research institution with strict quality-control procedures. Using microscopy, we were able to detect parasite densities as low as 2 parasites/µL of blood; it is estimated, however, that in an average health care facility with standard microscopy, the detection threshold would be 50–100 parasites/µL of blood (*30*,*31*). In some sites, the lower prevalence by PCR, compared with RDT, may be due to persistent antigenemia from past infections in the absence of current parasitemia.

The overall dynamics of infection across the 3 countries suggests that the period of protection against malaria may be the first 3 months of life, and thereafter the odds for malaria rise increasingly by age. Our findings therefore provide evidence that the period of perinatal protection may be shorter than 6 months and that the 0- to 6-month-old age group is not a homogenous group in terms of malaria susceptibility. Thus, the challenge is that young infants are not adequately protected against malaria because of their limited coverage by current preventive strategies, such as seasonal malaria chemoprevention and intermittent preventive treatment during infancy, which are not widely implemented. This inadequate coverage is critical because our findings show that young infants can be affected by malaria and subsequently become anemic, which would also potentially increase their vulnerability to other pathogens (*32*). Other interventions, such as the RTS,S/AS01 malaria vaccine, which will soon be registered for use, resulted in modest protection against clinical malaria in this age group and did not have any effect on preventing anemia (*33*).

In conclusion, the prevalence of malaria is sizeable among young infants living in malaria-endemic countries. This problem must be addressed through the development of adequate pediatric drug formulations, targeted preventive interventions, and treatment guidelines for young infants.
